# Field Measurement of the Dynamic Interaction between Urban Surfaces and Microclimates in Humid Subtropical Climates with Multiple Sensors

**DOI:** 10.3390/s23249835

**Published:** 2023-12-14

**Authors:** Min-cheng Tu, Wei-jen Chen

**Affiliations:** 1 Department of Civil Engineering, National Taipei University of Technology, Taipei City 106, Taiwan; 2 Undergraduate Program of Vehicle and Energy Engineering, National Taiwan Normal University, Taipei City 106, Taiwan; kylechen@ntnu.edu.tw

**Keywords:** field measurements, impervious, permeable, pervious, microclimate, multiple regression, urban heat island

## Abstract

Forcing pathways between urban surfaces (impervious and pervious pavers) and near-surface air temperature were measured and investigated with a network of multiple sensors. Utilizing field data measured between April 2021 and May 2022, and assuming that the influential variables follow the basic heat-transfer energy-balance equations, multiple regression-based statistical models were built to predict the surface temperature and near-surface air temperature (0.05 m, 0.5 m, 1 m, 2 m, and 3 m) of one impervious paver site and one pervious paver site in Taipei City, Taiwan. Evaporative cooling was found to be more influential on the pervious paver with a statistically significant influence on the microclimate up to 1.8 m (and up to 0.7 m for the impervious paver), using in situ data with an ambient air temperature higher than 24 °C. The surface temperature is mainly affected by solar shortwave radiation and ambient air temperature. As for near-surface air temperature, ambient air temperature is the most influential factor, followed by surface temperature. The importance of surface temperature indicates the influence of upwelling longwave radiation on the microclimate. The predictive equations show that pervious surfaces can help cities with hot and humid climates fight the changing climate in the future.

## 1. Introduction

Compared to the preindustrial world, the global temperature has increased by 1.19 °C, as of 2020 [1]. The temperature is expected to keep rising, and by 2100, the global temperature could undergo another hike of 1.1–5.4 °C [2]. On a global scale, higher global temperature increases the occurring frequency of heat waves, affects water availability, induces extreme precipitation distributions (generating floods and droughts), impacts biodiversity and ecosystems, decreases food supply, and could raise sea levels by 25–30 cm by 2050 [3,4]. 

On a local scale, heat waves and urban heat islands have profound synergies [5]. The impact of heat waves is intensified in cities due to urban heat islands. In cities, anthropogenic heat sources (cars, air conditioners, etc.) generate heat, which is difficult to dissipate from the canyons formed by tall buildings [6,7].Additionally, urban spaces are mainly composed of man-made impervious surfaces such as roofs and roads, which often show a higher surface temperature and thus affect the urban microclimate. The combination of hot summer temperatures and urban heat islands has made the urban environment unbearable, if not hazardous [8]. A small decrease in air temperature by 1–2 °C could reduce heat-related death by 32–69% [9]. Higher urban air temperatures also spur buildings to consume more electricity to power air conditioners [10]. Because more than half of the global population is living in cities now [11], this is no longer an isolated local problem, but a global one.

A few solutions can mitigate such an issue. Urban greening and water bodies are probably the most effective strategies [12,13]. Urban greening provides multiple benefits, including shading and cooling through evapotranspiration, while water bodies exhibit high thermal capacity and evaporation. In ultra-urban areas where all precious space is dedicated to buildings, roads, or pedestrian space, the manmade pervious (also called porous or permeable) surface is considered an attractive alternative [14]. The proportion of fine particles is reduced on the pervious surface to contain porous space allowing runoff infiltration [15]. Because water has a high heat capacity and latent heat of evaporation, the pervious surface is expected to have a lower surface temperature [16], thus lowering the near-surface air temperature. In the current paper, the term “near-surface” is defined as the space that is enough close to the ground to be highly affected by variations in ground temperature.

Based on such an expectation, many studies have focused on the influence of urban surfaces on urban microclimates. Chatzidimitriou and Yannas [17] measured the surface and microclimate temperatures at six urban locations with different surface materials and reported a few interesting findings. First of all, they found that a surface with higher albedo has a lower surface temperature, but the microclimate temperature (taken 1.1 m above ground) is not necessarily lower. Secondly, surface materials with higher heat capacity have lower surface temperatures and can have lower near-surface air temperatures. Finally, the porous material reduces the surface temperature. They also found that a grass surface under tree-canopy shade significantly reduces the microclimate temperature by 2 °C. Despite these important findings, Chatzidimitriou and Yannas only collected data for a short period of 11 days and did not provide quantitative explanations of the forcing between surface temperature and near-surface air temperature. Similarly, Yaghoobian and Kleissl [18] investigated the effect of reflective pavements on building energy use.

Focusing on urban microclimates, Dimoudi et al. [19] used a portable weather station with a height of 1.8 m to record near-surface microclimate parameters. Yaghoobian et al. [20] investigated the thermal effects of artificial turf on the urban environment. Using data collected 1.5–2 m above ground over 12 days, Huang et al. [21] investigated the influence of four different land covers on urban heat islands. Alternatively, some researchers chose to focus on the dynamics of surface temperature. Chen et al. [22] conducted lab experiments to compare the surface temperature of pervious and conventional concrete slabs and concluded that lower surface temperatures are associated with higher surface moisture, depending on the water evaporation rate. Li et al. [23] conducted a similar experiment to study the interaction between porosity, permeability, evaporation rate, and surface temperature of pervious pavement materials. Even though microclimate and surface temperature have received separate attention from different researchers, there have been very few studies focusing on quantifying the interaction between urban surfaces (particularly pervious surfaces) and near-surface microclimate air temperatures. Wang et al. [24] showed that sprinkling can reduce the surface temperature and near-surface air temperature of ceramic porous brick and open-graded permeable concrete by 10 °C and 1 °C, respectively. Without frequent wetting (such as under drought conditions), the cooling effect of permeable concrete is reduced drastically. Recently, Amani-Beni et al. [25] installed a bike-mounted weather station to collect microclimate attributes and environmental conditions, but the data collection was limited to only 1.5 m and the focus was the spatial distribution of the heat island effect. Utilizing hourly observations of air temperature at 1.5 m and 3.0 m, infrared surface temperature, vapor pressure, incoming solar radiation, and vector wind velocity, Stoll and Brazel [26] performed regression analyses. They found that surface temperature is the primary factor affecting the microclimate.

Metrics like PET (physiological equivalent temperature) might be more appropriate to measure how pedestrians actually feel, but PET requires conversions based on specific weather conditions and specific conditions of clothing and physical activities of pedestrians [27]. Therefore, the current study focused on the generic indicator of microclimate air temperature to be comparable with the cited work above.

As summarized above, the literature provided some qualitative studies on the influence of surface materials on microclimate but did not investigate the forcing pathways between the surface and near-surface microclimate air temperature. In addition, most existing microclimate studies contained only short periods of data or did not contain complete vertical temperature profiles to fully show the variation in the microclimate at different altitudes. The current study was designed to fill the gaps. With data from multiple sets of sensors, the current study predicted surface temperature and near-surface air temperature at different altitudes and tried to explain the different forcing pathways between the surface and near-surface air temperature, based on complete (up to 3 m) vertical temperature profile measurements spanning a whole year.

## 2. Site Description

The experimental sites with approximate coordinates (25.042° N, 121.533° E) are at a section of the newly installed pervious sidewalk in the center of Taipei City. Taipei City is located in northern Taiwan, and has a humid subtropical climate. The annual mean temperature is around 21 °C and the annual rainfall depth is around 2400 mm [28]. Except for the outlying mountains, the city itself has never received snow.

**Figure 1:**
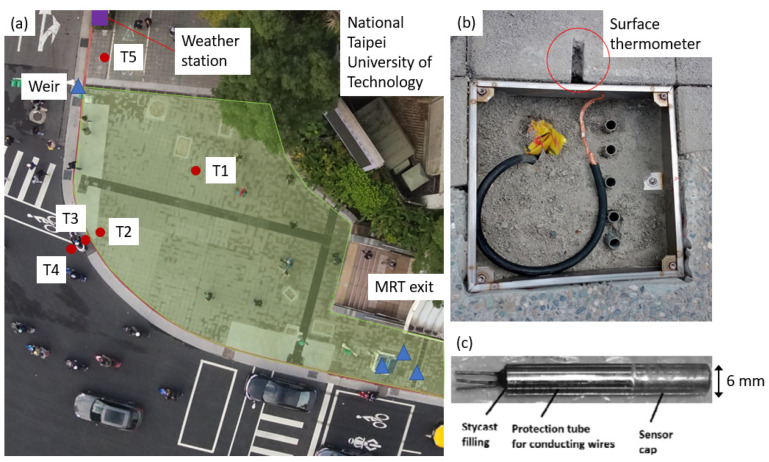
(**a**) Aerial photo of the experimental sites with locations of underground thermometers (red circles, T1-T5), runoff gages (blue triangles, not used in this study), and the weather station (purple square) marked, (**b**) scheme of surface and underground temperature monitoring before sensor instrument installation with the location of surface thermometers marked, and (**c**) close-up view of PT-1000 sensor used for underground and surface temperature measurements (Adapted from Ramalingam et al. [29]).

The pedestrian space along Zhongxiao E. Road, one of the city’s main boulevards, underwent major improvements in December 2020. The pervious sidewalk was one of the highlights of the improvement project, and a large monitoring project was embarked on to monitor its hydrological and thermal performance. The results presented in the current study were only a small part of the monitoring project. Several sets of monitoring instruments were installed during paver construction, including underground runoff observation weirs, a comprehensive weather station set, and surface/underground thermometers, as shown in Figure 1. Data recording using on-site instruments began in March 2021 and is continuing.

In Figure 1a, the green-shaded area contains the pervious pavers and the rest of the sidewalk consists of the impervious pavers. The preexisting impervious pavers were formed by precast high-pressure concrete blocks with dimensions of 30 cm × 30 cm × 6 cm. The mean mechanical strength of the impervious pavers is about 95 MPa. The newly installed pervious pavers (the green-shaded area in Figure 1) are precast concrete blocks of the same dimensions with a mean strength of approximately 65 MPa. The pervious paver has a nominal porosity of approximately 25% and a mean permeability coefficient (*K*_20_) of about 1.1 × 10^−1^ cm/s (personal communication with Jinhuang Construction, Taipei City, Taiwan).

Five temperature-monitoring points were installed: T1, T2, T3, T4, and T5, with T1 and T2 at the pervious site. At each of T1, T2, T3, and T5, five PT1000 thermometers (WIKA Instruments, Taoyuan, Taiwan) were installed at different depths (0.09 m, 0.17 m, 0.24 m, 0.32 m, and 0.36 m) through shafts visible in Figure 1b, and one was installed at 0.015 m depth through a surface notch (indicated with a red circle in Figure 1b). The specification of the PT1000 thermometer (Figure 1c) is provided in Table 1. T4 had only the surface temperature measured.

The current study utilized only surface temperature (i.e., 0.015 m depth) data from T1 (pervious paver) and T5 (existing impervious paver). Points T1 and T5 were chosen to be as far away from surrounding objects as possible. T1 is about 6 m and T5 is about 1.5 m away from the nearest tree planter. The selected location of T5 was the result of compromises of many practical considerations. Even though T5 is in close proximity to the pervious paver, the distance (~2.5 m) is still on par with the spacing used by other researchers [24].

**Figure 2:**
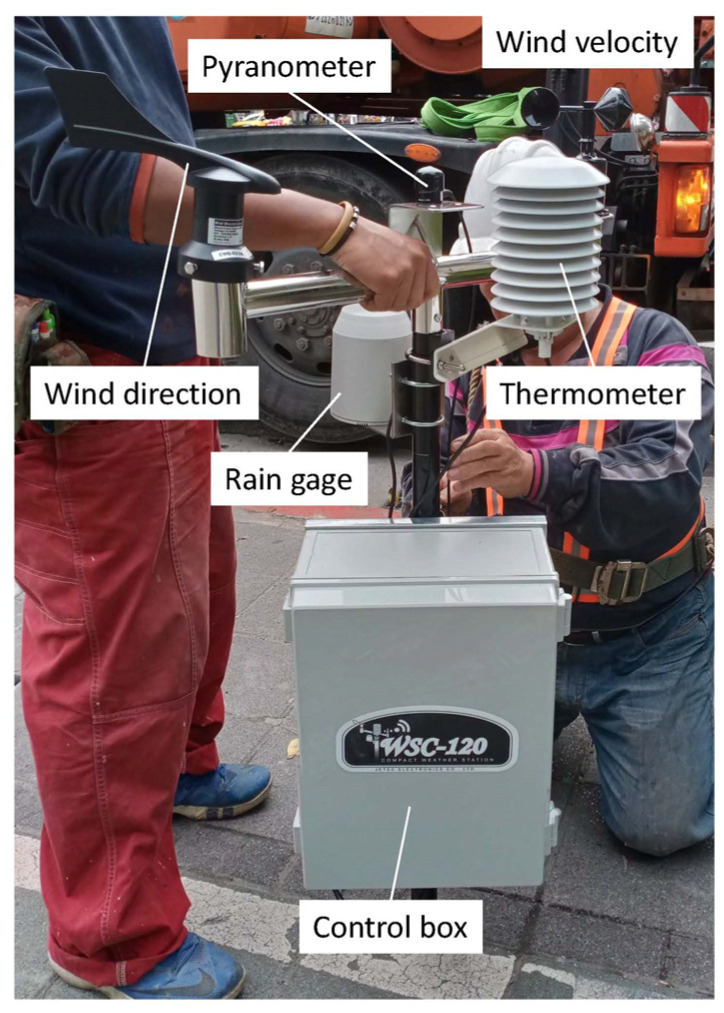
Close-up view of the weather station sensor set.

The weather station set (Figure 2) is composed of a WSC-120 weather station (Jetec Electronics, Taichung, Taiwan) and a JSQ-214 pyranometer (Apogee Instruments, Logan, UT, USA) installed at a height of 8 m above the ground. Specifications of the weather station and pyranometer are provided in Table 1. They are represented by a purple square in Figure 1a. Air temperature, relative humidity, solar radiation (in the form of irradiance), wind direction, and wind speed data were collected on-site. The data collected from the weather station were considered by the current study as the ambient conditions without being affected by either the pervious or impervious surface. Although the ambient condition can vary depending on location and altitude, the data from the weather station were considered the “nominal” ambient conditions in this study. Both the weather station and the surface thermometers are part of an IoT (Internet-of-things) network mentioned below.

The blue triangles in Figure 1a represented the locations of triangular weirs installed underground in stormwater inlets or sewers to monitor surface runoff rates, utilizing the relation between depth and flow rate [30]. Since hydrology is not the focus of the current study, only the locations of the weirs are provided. The albedo of the pervious and impervious paver was measured by two coupled SP-214-SS pyranometers (Apogee Instruments, Logan, UT, USA) as 0.17 and 0.15, respectively. The pyranometers were shielded at the edge of the field of view to create a limited ground measuring area with a diameter of approximately 3.5 m. The specification of the SP-214-SS pyranometer is provided in Table 1.

All the sensors above are part of an IoT (Internet-of-things) network for easy data monitoring, collection, and synchronization. The system was built by Fenri Co., Ltd. (New Taipei City, Taiwan). Data collected from all instruments are sent (through cables) to the IoT control panel located beneath the weather station at the ground level. The IoT control panel utilizes the LPWAN (Low-Power Wide-Area Network) protocol to transmit collected data to a cloud server. Real-time data can be viewed and downloaded from the server at https://ntut.fenri.com.tw/zhongxiao/ (accessed on 4 December 2023).

The sites are on the northern side of a 40 m road and the surrounding buildings are not very tall, thus receiving ample sunlight throughout most of the day. The buildings in the south and southwest of the site are both 15 stories high, and the building in the direct west is 22 stories high. Based on field observations, T1 and T5 both received direct sunlight from noon to 5–6 pm. Only data points when T1 and T5 both receive direct sunlight were included in the analysis of the current study.

Besides the National Taipei University of Technology, most zoning in the proximate area is either commercial or mixed-use commercial. The sites frequently receive heavy pedestrian traffic because of the proximity to an MRT (subway) exit, the university, and a wholesale market of electronic gadgets.

**Figure 3:**
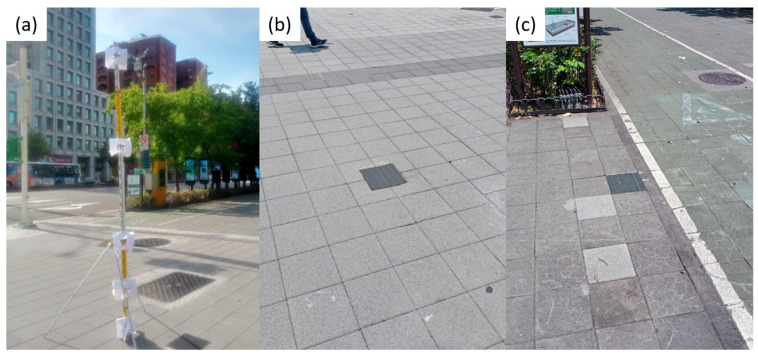
Near-surface air temperature measurement sensor setup and locations: (**a**) the apparatus of near-surface air temperature measurement (white heat shields visible); (**b**) the vicinity of the measurement point at the pervious site; and (**c**) the vicinity of the measurement point at the impervious site, where the dark covers in the center of (**b**,**c**) house underground thermometer shafts.

The current study focused on surface temperature and near-surface air temperature. Surface temperature was measured by on-site instruments as shown in Figure 1. However, establishing fixed on-site instruments to measure near-surface air temperature was difficult for such sites with significant pedestrian traffic. A mobile apparatus (Figure 3a) was created to house five THD-8 electronic thermometers (Jetec Electronics, Taichung, Taiwan) at altitudes of 0.05 m, 0.5 m, 1 m, 2 m, and 3 m. The specification of the THD-8 thermometer is provided in Table 1. The highest measurement point was at 3 m because Taleghani and Berardi [31] found that the temperature regulation by the ground surface becomes insignificant above 3 m. Heat shields above and below the thermometers were installed to ensure all thermometers are not directly exposed to sunlight from above and surface heat radiation from below. The apparatus was moved to point T1 on the pervious site (Figure 3b) and point T5 on the impervious site (Figure 3c) every afternoon (except for holidays and rainy days), between April 2021 and May 2022, to measure in-situ near-surface air temperature, because the sites are partly shadowed in the morning. Measurements followed the protocol that the apparatus must stay close to but not cover the surface thermometer at each measurement point for at least 10 min before data recording to ensure temperature equilibrium. The measurements should be performed when the weather condition is steady between noon and sunset to make sure both sites and the weather station receive the same amount of sunlight (the sites are under tree shadow in the morning) during data collection. Before deployment in the field, all thermometers were placed together in an air-conditioned room for 30 min to confirm consistent temperature readings (within a bracket of ±0.3 °C) of all the thermometers. The manually measured microclimate data were saved in a Google Sheet spreadsheet on the cloud to be shared by the research team.

## 3. Methodology

### 3.1. Effect of Surface Water Content

In this study, statistical modeling was performed to examine the effect of surface moisture content on near-surface air temperature. Surface moisture content was represented by two parameters: the rate of water entering the pavement (antecedent rainfall depth) and the rate of water leaving the pavement (water evaporation rate). Water percolation into the native soil was negligible because all recorded antecedent storms for the 84 in situ measurements had a rainfall depth of less than 0.15 m, the approximate depth of water that can be held by the structure of the pervious site. Antecedent ambient air temperature was used as a surrogate of the water evaporation rate, with a higher mean temperature being the main factor to increase water evaporation from the surface [32].

A total of 30 statistical models (15 for the pervious site and 15 for the impervious site) were built to examine the effects of surface moisture content on the microclimate at 5 different altitudes (0.05 m, 0.5 m, 1 m, 2 m, and 3 m) for each of the antecedent timeframes (24 h, 48 h, or 72 h). Linear multiple regression models were built for each timeframe using the near-surface air temperature as the independent variable and antecedent rainfall depth, mean air temperature, and their interaction term as the dependent variables.

### 3.2. Surface Temperature

Besides surface moisture content, many studies identified surface temperature as the key factor influencing the microclimate [25,26,33,34]. The surface energy balance equation [35] is delineated in Equation (1) below:

(1) $$G_{n}=R_{n}-SH-LH=\left(\left(1-a\right)SWD+LWD-LWU\right)-SH-LH$$

where $G_{n}$ is net ground heat flux, $R_{n}$ is net radiation, SH is sensible heat flux, LH is latent heat flux, a is surface albedo, SWD is downwelling shortwave (solar) radiation, LWD is downwelling longwave (thermal) radiation, and LWU is upwelling longwave radiation. Longwave radiation terms LWD−LWU can be represented by Equation (2), and SH can be represented by Equation (3) below [35,36]:

(2) $$LWD-LWU=\varepsilon \sigma \theta^{4}-\varepsilon_{s}\sigma \theta_{s}^{4}=1.24{\left(\frac{e}{\theta }\right)}^{0.14286}\cdot \sigma \theta^{4}-\varepsilon_{s}\sigma \theta_{s}^{4}$$

(3) $$SH\propto \rho C_{p}C_{h}u(\theta_{s}-\theta )$$

In Equation (2), ε is air emissivity, σ is the Stefan-Boltzmann constant, θ is air temperature (°K), $\varepsilon_{s}$ is surface emissivity, $\theta_{s}$ is surface temperature (°K), and e is water vapor pressure. In Equation (3), ρ, $C_{p}$, $C_{h}$, and u are air density, the specific heat capacity of air at constant pressure, turbulent exchange coefficient for sensible heat, and wind speed, respectively.

To tackle the fluctuation of surface temperature, two statistical models were created for the surface temperature of the pervious and impervious sites, respectively. Initial candidate variables in Table 2 were chosen based on Equations (1)–(3) above. Wind speed, ambient air temperature, and solar radiation were chosen because they are major terms in the equations and among the available data. Theoretically, 0.05 m air temperature is more appropriate than the ambient air temperature. Table 2 included the ambient air temperature, rather than 0.05 m air temperature, because that ambient air temperature was continuously available, which was not the case for 0.05 m air temperature. Utilizing ambient air temperature maximized the applicability of the derived models. The ambient air temperature and 0.05 m air temperature are highly correlated (correlation is 0.92 for the pervious site and 0.93 for the impervious site), so utilizing ambient air temperature would not considerably reduce the model accuracy.

The interaction term of x-hour antecedent mean ambient air temperature ($T_{x-h}$) and x-hour antecedent rainfall depth ($R_{x-h}$) represented surface moisture content, which contributed to the water available for evaporation and can be directly related to the latent heat flux (LH in Equation (1)). The value of x was decided based on the time frame(s) that showed a significant relationship between surface moisture and microclimate (i.e., the result from Section 3.1).

Water vapor pressure e (which can be derived from relative humidity and air temperature [37]) was not chosen because the vapor pressure forms a non-linear relation with the net longwave radiation, based on Equation (2). Data from this study showed a low correlation between vapor pressure and surface temperature (0.19 for the pervious and 0.25 for the impervious sites). Many empirical models also made the same call by excluding vapor pressure or relative humidity from statistical models [38]. Surface albedo a was not chosen mainly because the statistical models were derived independently for each site. Besides, the difference in albedo between the two sites was considered too small to show a meaningful influence on surface temperature (the correlation between albedo and surface temperature is only 0.15).

The models were created based on minimal AIC (Akaike Information Criterion). At each step of the forward variable selection, only the variable that decreased the most AIC entered the model until the lowest AIC was reached. A model with minimal AIC explains the most variation with the least number of predictive variables. AIC is a commonly used criterion and is often superior to other selection methods [39]. The VIF (Variation Inflation Factor) of each selected variable was monitored to keep multicollinearity in check [40].

### 3.3. Near-Surface Microclimate Temperature

Near-surface air temperature is part of the energy transfer between surface and microclimate, thus it is also governed by Equations (1)–(3). Utilizing the same approach, multiple regression models can also be created for microclimate temperature at 0.05 m, 0.5 m, 1 m, 2 m, and 3 m. The initial candidate variables were shown in Table 3. Identical to Section 3.2, x was decided based on the time frame(s) that showed a significant relationship between surface moisture and microclimate (i.e., the result from Section 3.1). The variables contained in Table 3 are very similar to those in Table 2, with Table 3 containing one additional predictive variable, the surface temperature ($T_{surf}$), because the near-surface air temperature is affected by more variables compared to the surface temperature according to Equations (1)–(3). Like the ambient air temperature, all variables (including the surface temperature $T_{surf}$) used in the derivation of the statistical models of this section were recorded continuously for the experimental sites. 

## 4. Data Analyses Results

### 4.1. Data Summary

This study focused on data collected in hot weather, as the heat island effect is more prominent in summer [41]. Data points with ambient air temperature higher than 24 °C were included. The criterion of 24 °C was chosen subjectively as it is the lower bound of the monthly maximum temperature in the summer months (May–October) in Taipei City [28]. 

Data collection using the mobile apparatus (Figure 3a) began in April 2021 and ended in May 2022, but the data used in the current study were truncated at the end of March 2022 to keep the duration exactly one year. A total of 84 measurements of near-surface air temperature were selected (along with the corresponding surface temperature and ambient weather parameters) in the following analyses, which were summarized in Table 4 and Table 5. In Table 4 and Table 5, the wind speed, air temperature (ambient), relative humidity, and solar radiation data were from the weather station (Figure 2), the surface temperature was from the surface thermometer (Figure 1b,c), and near-surface air temperature (0.05 m, 0.5 m, 1 m, 2 m, and 3 m) was from the mobile apparatus (Figure 3a).

**Figure 4:**
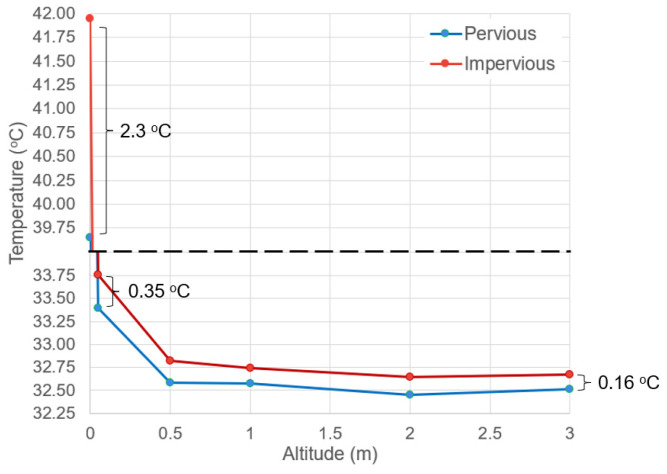
Summary of mean surface and near-surface air temperature at different altitudes of both paver types with a dashed line showing the skipped range of temperature.

The mean temperatures of the surface (altitude = 0), 0.05 m air, 0.5 m air, 1 m air, 2 m air, and 3 m air at both sites were plotted in Figure 4. Figure 4 showed that the mean surface temperature of the impervious paver was indeed significantly hotter than that of the pervious paver (*t*-test *p*-value = 0.001). Nevertheless, such a significant temperature difference did not exert an influence of similar magnitude on the microclimate. Even though the mean air temperature at the impervious site at all altitudes was still higher than that of the pervious site, such a difference did not pose statistical significance (all *t*-test *p*-values > 0.05). 

### 4.2. Effect of Surface Water Content

Among the 30 statistical models, only models considering the 24 h antecedent period showed statistical significance, and were extracted and shown in Table 6. Temperature data other than the measured altitudes of 0.05 m, 0.5 m, 1 m, 2 m, and 3 m were interpolated. For the pervious site, the 24 h interaction terms significantly contributed to near-surface air temperatures for the majority of altitudes, up to approximately 1.8 m. Such a relationship was also found for the impervious site with a shorter effective altitude, up to approximately 0.7 m. Only the interaction term is statistically significant (neither main effect is significant), indicating a “crossover” interaction [42]. Only the two main effects (rainfall depth and mean temperature) together have explanatory power on near-surface air temperature. The overall effect of either main effect alone would be offset by the effect of the interaction.

### 4.3. Surface Temperature

**Figure 5:**
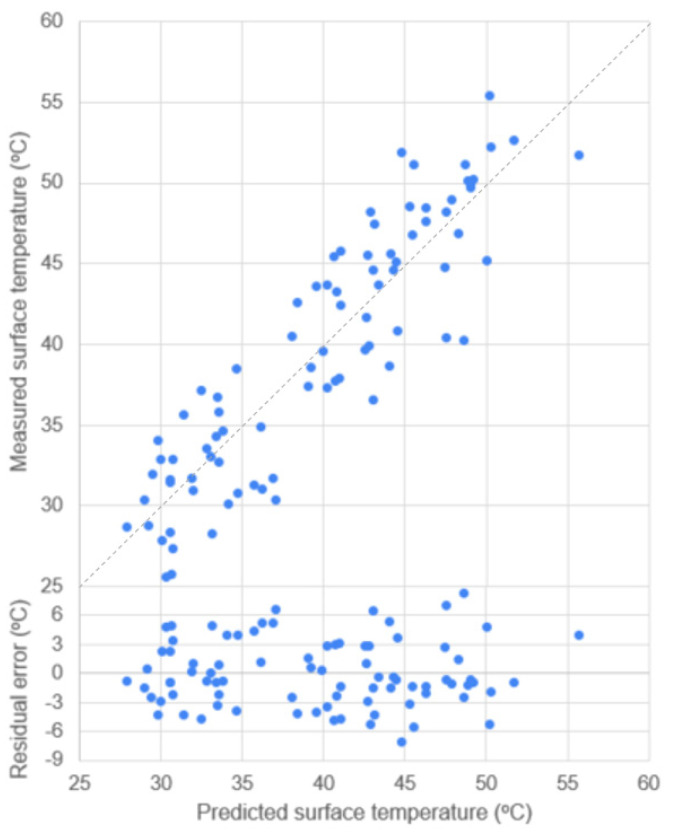
Scatterplot of data points of predicted vs. measured surface temperature and residual error for pervious paver with R^2^ = 0.81 (dash line is 1:1).

**Figure 6:**
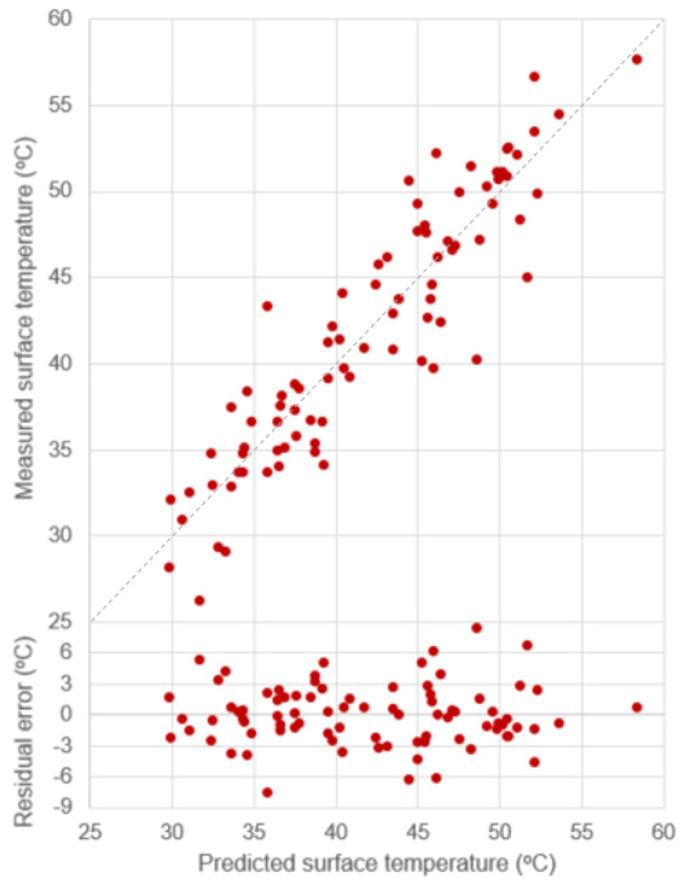
Scatterplot of data points of predicted vs. measured surface temperature and residual error for the impervious paver with R^2^ = 0.84 (dash line is 1:1).

The predictive models for pervious and impervious paver surface temperature are derived and presented in Equation (4) (R^2^ = 0.81) and Equation (5) (R^2^ = 0.84), respectively. The *p*-value and 95% CI range of coefficients are reported in Table 7. The scatterplots between predicted and measured surface temperature for pervious and impervious pavers with the distribution of residual error are provided in Figure 5 and Figure 6, respectively.

(4) $$T_{s,pervious}=5.01+1.22\cdot T+12.56\cdot SR-0.27\cdot T_{24h}-0.043\cdot R_{24h}-0.086\;\cdot \left({(T}_{24h}-28.27)\cdot (R_{24h}-3.63)\right)$$

(5) $$T_{s,impervious}=1.22+1.25\cdot T+11.37\cdot SR-0.080\cdot T_{24h}+0.015\cdot R_{24h}-0.071\;\cdot \left({(T}_{24h}-28.31)\cdot (R_{24h}-3.21)\right)$$

### 4.4. Near-Surface Microclimate Air Temperature

**Figure 7:**
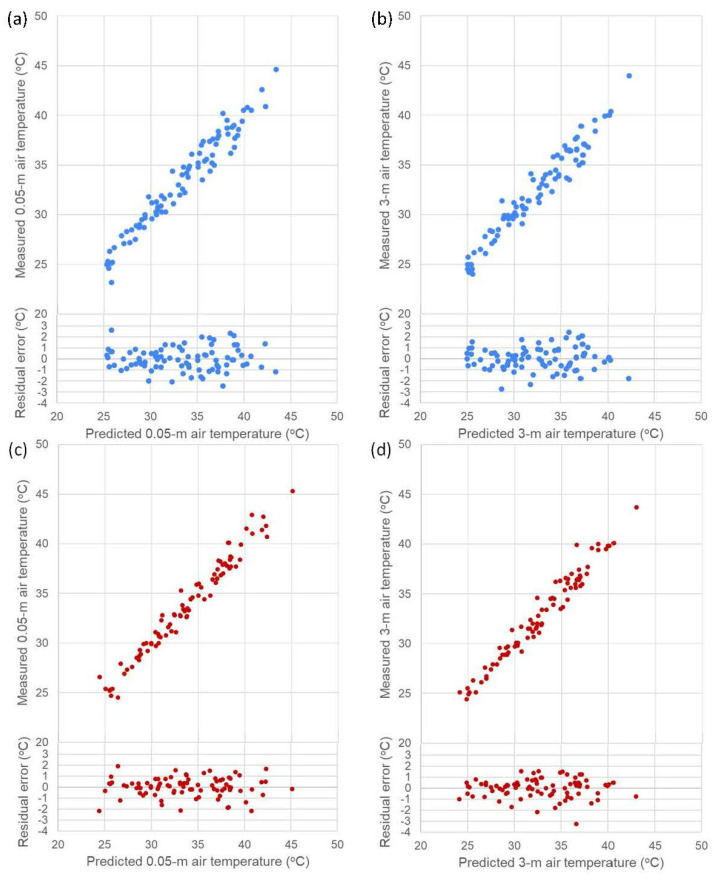
Scatterplots of data points of predicted vs. measured near-surface air temperature (dash lines are 1:1) for (**a**) the pervious site at 0.05 m altitude, (**b**) the pervious site at 3 m altitude, (**c**) the impervious site at 0.05 m altitude, and (**d**) the impervious site at 3 m altitude.

The derived models for the near-surface air temperature at the pervious and impervious sites are described in Table 8 and Table 9, respectively. Selected scatterplots (0.05 m and 3 m) of predicted vs. measured air temperatures with the distribution of residual error are provided in Figure 7 as examples for visual assessment.

## 5. Discussion

From the data summarized in Figure 4, the result showed a clear difference for the mean surface temperature, but not the mean near-surface air temperature. Further statistical analyses showed that only the interaction term of antecedent rainfall depth and antecedent mean air temperature can explain the variation in near-surface air temperature (Table 6). Similar observations were also made by other researchers [43] that the difference in mean near-surface air temperature at different sites is often very small, but surface soil moisture content can be found to influence the microclimate. Such explanative power diminishes with increasing altitude and becomes insignificant at about 1.8 m for the pervious site and about 0.7 m for the impervious site. It was posited in this study that the interaction term can qualitatively represent the magnitude of surface moisture content. The statistical insignificance of the main effects and significance of the interaction term suggested that the surface moisture content is not simply decided by either rainfall depth or potential evaporation alone, but is a complicated balance between the two. The surface moisture content of the pervious paver has a higher influence on the microclimate compared to that of the impervious paver, possibly due to the larger storage space for water and higher evaporation rate of the pervious surface [44]. The result also showed that such a qualitative relation is valid for an antecedent time frame of 24 h. Qin and Hiller [16] also found a similar time frame for the duration that infiltrated water stays in pervious concrete before being completely evaporated on a typical hot summer day.

Surface temperature, which is a very important factor influencing microclimate temperature [25,26,33,34], was significantly affected by solar radiation for both pervious and impervious surface temperature (both *p*-value < 0.0001) based on Table 7. This is physically sound as solar radiation (SWD in Equation (1)) is the only energy input considering the air–ground domain as a closed system. Near-surface air temperature (surrogated by ambient air temperature in the models) is the second important term (*p*-value = 0.0005 for pervious surface and *p*-value < 0.0001 for impervious surface). The air–surface temperature difference directly affects the sensible heat flux (SH in Equation (3)) and is related to the net longwave radiation flux. The higher the ambient air temperature, the smaller the flux of sensible heat and the higher the downwelling longwave radiation, which results in a higher surface temperature. However, wind speed u was not found to be a significant predictor variable. This was possibly caused by all manual in situ measurements being performed when weather conditions were steady, and most of such conditions were windless, thus reducing the effect of wind speed. Another possibility is that urban geometry significantly reduces the influence of wind due to the sheltering effect [45]. Because of the low influence of wind speed, the pathway of sensible heat could be less important in the urban setting. Although the ambient air temperature was considered to enhance evaporative cooling (latent heat LH) of the surface [46], overall it poses a positive influence on surface temperature according to this study.

The most important finding of this part was confirming the role of surface moisture content in regulating surface temperature. Surface moisture content (represented by the interaction term of antecedent rainfall depth and mean air temperature) is a very significant influencer for both pervious (*p*-value = 0.0004) and impervious (*p*-value = 0.0012) surface temperature. Water provides a high heat capacity and an evaporative cooling effect [16]. Both pervious and impervious pavers showed such an effect, implying that water stored in crevices and surface ponding of the impervious pavers also contributed to surface cooling, but the pervious paver still showed stronger surface cooling based on the coefficients in Equations (4) and (5) (−0.086 vs. −0.071), possibly due to its larger water storage space.

**Figure 8:**
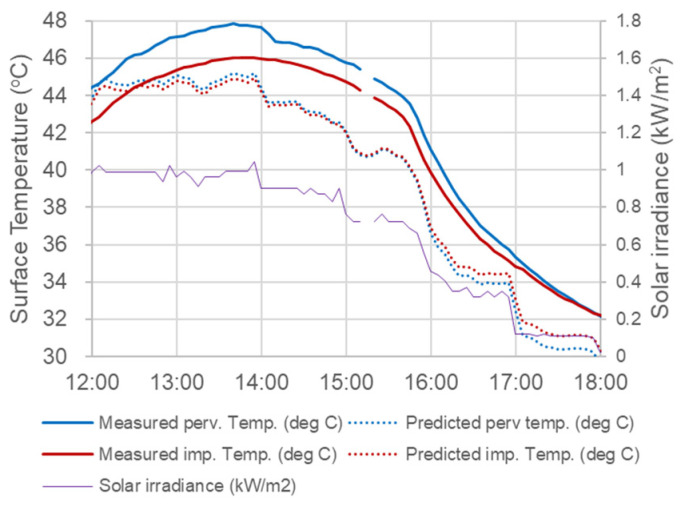
An example of the higher surface temperature of pervious paver with high solar irradiance and no surface moisture content.

The predictive equations for surface temperature showed an interesting phenomenon. Coefficients of solar radiation (SR) are much higher in Equation (4) (pervious) compared to those in Equation (5) (impervious), implying a quicker response of surface temperature caused by solar radiation for pervious pavers when dry. Figure 8 showed one field example from the data recorded on 7 April 2021. In the early afternoon (12:00–15:00), both the measured and predicted surface temperature of the pervious paver was higher. The temperature difference was reduced and inverted with lower solar irradiance after 15:00–16:00. A similar phenomenon was also observed by several other researchers [22,47] and was explained by the lower heat capacity of the pervious surface because of the lower mass density [47]. The predicted temperature in Figure 8 was systematically underestimated, which was probably caused by the fact that the air temperature in April was close to the lower boundary (24 °C) of data selected for this study.

As mentioned before, near-surface air temperature was highly correlated with ambient air temperature, which explained why ambient air temperature (T) is the most significant predictor variable in Table 8 and Table 9. Surface temperature ($T_{surf}$) is the second most significant influencing factor. Based on Equations (1)–(3), the surface temperature directly controls the magnitude of sensible heat flux (SH) and the amount of upward longwave radiation (LWU). Higher surface temperature increases the influence of both pathways to heat near-surface air. However, the longwave radiation component should be dominant as wind speed is not a significant influencing variable, thus minimizing the influence of the sensible heat flux as discussed before. In general, both the significance (indicated by *p*-value) and the magnitude of influence (indicated by coefficients) were found to decrease as the altitude increases, indicating that the warming effect decreases as the distance from the surface increases. 

Solar radiation (SR) is also significant (but not as significant as T or $T_{surf}$) in the statistical models. The significance of solar radiation on near-surface air temperature increases as altitude increases, implying that solar radiation reflected from the ground does not heat air effectively. The observed trend can be explained by the fact that higher positions receive relatively more skylights compared to lower positions in urban canyons [48].

An interesting contrast between Table 8 and Table 9 is that $T_{24h}$ (24 h mean ambient air temperature) and $R_{24h}$ (24 h accumulated rainfall depth) are not significant predictor variables for the near-surface air temperature at the pervious site, but they are significant for that of the impervious site (at most altitudes). Another observation is that the interaction of $T_{24h}$ and $R_{24h}$ is not statistically significant for both sites. It is hypothesized that ambient temperature and cumulative rainfall have separate and independent effects (instead of representing surface moisture content when considered together) on near-surface air temperature. 

It was hypothesized in this study that long-term ambient air temperature ($T_{24h}$) affects cumulative water evaporation over the antecedent 24 h period. Higher long-term air temperature means higher cumulative evaporation, which reduces surface moisture and decreases surface emissivity noticeably [49]. Lower surface emissivity reduces the upward longwave radiation from the surface; thus, a higher $T_{24h}$ has a net negative forcing on near-surface air temperature at the impervious site. Such a phenomenon was not observed at the pervious site because its surface moisture might be regulated through capillary exaction of water stored in the paver structure [44]. Such a hypothesis still needs to be proven by future experiments. 

Higher $R_{24h}$ potentially allows more evaporation, causing higher near-surface water vapor pressure. Higher vapor pressure (e in Equation (2)) increases the air emissivity, thus elevating downward longwave radiation. This is why water vapor is a strong greenhouse gas [50]. Since the greenhouse effect can vary locally according to the adjacent environment [51], this study hypothesized that varying vapor pressure can also affect the local microclimate. Theoretically, such a relationship should be prominent at the pervious site, where an ample water supply for evaporation is available. However, $R_{24h}$ showed significance to near-surface air temperature at the impervious site instead of the pervious one. This confusing phenomenon can be explained by Gobel et al. [52]. They found that the impervious surface had higher net evaporation than the pervious surface did in summer, and attributed this reversal in evaporation levels to vegetation existing in the seam of the impervious surface. It should be made clear that the higher net evaporation was not from the surface but was transpiration by vegetation, which was particularly significant in summer. Like the site used by Gobel et al., the impervious surface of this study is also old with visible cracks and a neighboring a vegetation planter (Figure 3c). The measurement point was only about 1.5 m away from the planter. The higher water vapor pressure was potentially generated by not only transpiration of vegetation in seams of the impervious surface, but also established vegetation in the nearby planters, and was irrelevant to the paver properties. Please note that the multiple hypotheses above need to be proven by future experiments.

By comparing results from Table 6, Table 8, and Table 9, a superficial discrepancy arose. Surface moisture content (represented by $T_{24h}$ × $R_{24h}$) shows significant relations in Table 6, but such a relationship does not exist in either Table 8 or Table 9, while all three tables describe factors influencing near-surface air temperature. This was explained by the fact that Table 6 considers only the influence of $T_{24h}$, $R_{24h}$, and their interaction. Table 6 shows the net influence of surface moisture content on near-surface air temperature. On the other hand, Table 8 and Table 9 consider multiple factors, of which surface temperature is a major one. Because surface temperature is influenced by surface moisture content ($T_{24h}$ × $R_{24h}$ in Table 7) through evaporative cooling, the statistical models of near-surface air temperature excluded $T_{24h}$ × $R_{24h}$ from the final forms to remove redundancy.

**Figure 9:**
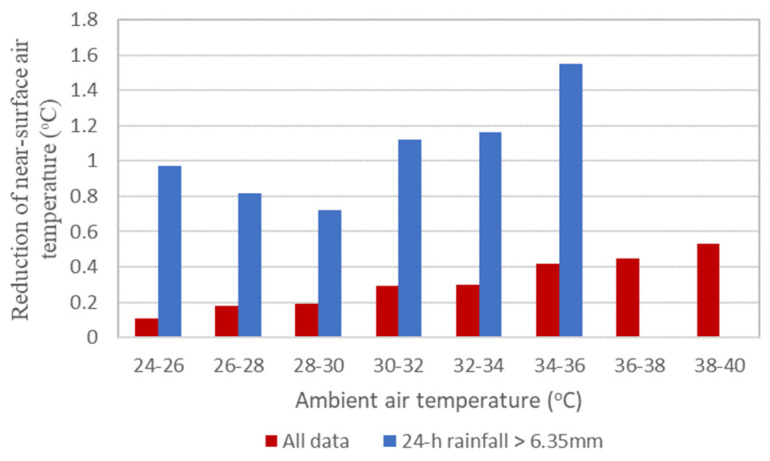
Reduction of near-surface air temperature by pervious surface for different ambient air temperatures.

The predictive models derived from this study provided a way to peek into how pervious paver affects the microclimate under the changing climate. Figure 9 provides a predicted near-surface air temperature (represented by air temperature interpolated for 1.5 m), calculated based on actual data between April 2021 and March 2022 for different ambient air temperature intervals. Figure 9 shows only very small reductions of near-surface air temperature by the pervious pavers for different ambient temperature ranges, with higher ambient temperatures exhibiting slightly higher temperature reduction effects. However, if only data with antecedent rainfall was chosen (blue bars), the reduction effect jumped up significantly (all *t*-test *p*-value < 0.05), with a reduction of almost 1.6 °C when the ambient temperature reaches 34–36 °C. The temperature reduction fluctuated between 24–30 °C because data entries with an ambient air temperature of 24–28 °C had much higher antecedent rainfall, thus biasing the results. Such a degree of temperature reduction is on par with the temperature reduction by grassy surfaces and tree canopies [17]. The pervious surface can provide ameliorating effects for hot and humid urban environments in the future.

## 6. Conclusions

Compared to the impervious paver, the pervious paver has a larger latent heat flux because its water storage sustains higher net evaporation. Although not quantified in this study, evaporation was considered the major cooling mechanism for urban surfaces, particularly for pervious pavement [46]. 

Caused by higher heat removal through the latent heat flux, the pervious paver often has a lower surface temperature. The comparatively higher surface temperature of the impervious paver generates higher (compared to the pervious paver) upwelling longwave radiation (LWU) and sensible heat (SH) fluxes to heat near-surface air. Wind speed showed little effect on both surface temperature and near-surface air temperature, implying that the sensible heat flux was not a significant pathway at the sites. 

This study also found that the higher water storage at the pervious site should have a higher combined effect on near-surface air temperature, as concluded in Table 6. The combined effect reaches approximately 1.8 m above ground at the pervious site, but only 0.7 m at the impervious site. However, the pervious pavers also can have a more rapid response to solar radiation input if the stored water dries out. Thus, a good implementation strategy is to avoid installing pervious pavers at locations exposed to direct sunlight.

There are a few points that can be improved in this study. The site is limited in size, so the measurement of near-surface air temperature at the impervious site was likely to be influenced by the surrounding vegetation and pervious paver. However, the size of the surrounding impervious paver and the proximity between sites are close to the experimental pads used by Wang et al. [24]. The data showed meaningful results, implying the heterogeneity of microclimates in urban environments [53]. Another possible issue is that the analysis focused on data collected in the afternoon (around 13:00–18:00), so the conclusions drawn from this study might not be representative of other situations. Finally, this study focused on air temperature only. Future studies can utilize composite indicators such as PET to investigate pedestrians’ heat stress under different microclimate conditions.

Urban space is the hub of livelihood for more than half of the world’s population. Improving the urban environment directly affects the lives of millions of people, if not billions. The preliminary predictions in Figure 9 show that the beneficial effects of pervious surfaces should particularly stand out in areas with humid climates where rainfall is ample and frequent. Assuming similar rainfall patterns in the future (neglecting the possible link between drought and climate change [54]), Taiwan, along with other tropical and subtropical countries, could benefit from a widespread installation of pervious surfaces in the fight against the changing climate.

## Figures and Tables

**Table 1:** Main parameters of the sensor instruments used in the current study.

PT1000 Surface Temperature Sensor		
Temperature	Range	−50–110 °C
	Accuracy	±1%
Sampling interval	5 min	
WSC-120 Weather Station		
Temperature	Range	0–60 °C
	Accuracy	±0.3 °C
Humidity	Range	0–100% RH
	Accuracy	±3% RH
Wind speed	Range	0–60 m/s
	Accuracy	± (0.3 + 0.03 × wind speed) m/s
Wind direction	Range	0–360°
	Accuracy	±2°
Rainfall	Accuracy	1 mm
Sampling interval	5 min	
JSQ-214 Pyranometer		
Field of view	180°	
Spectral range	410–655 nm	
Calibration uncertainty	±5%	
Sampling interval	5 min	
SP-214-SS Pyranometer		
Field of view	180°	
Spectral range	360–1120 nm	
Calibration uncertainty	±3%	
Sampling interval	5 min	
THD-8 Air Temperature and Humidity Sensor		
Temperature	Range	−20–80 °C
	Accuracy	±0.3 °C
Humidity	Range	0–100% RH
	Accuracy	±2% RH
Sampling interval	5 s	

**Table 2:** Initial predictive variables for input into the statistical models of pervious and impervious paver surface temperature.

Symbol	Description
u	Wind speed (m/s)
T	Ambient air temperature (°C)
SR	Solar radiation (in irradiance, kW/m^2^)
$T_{x-h}$	x-hour antecedent mean ambient air temperature (°C)
$R_{x-h}$	x-hour antecedent rainfall depth (mm)
$T_{x-h}\times R_{x-h}\;$ *	The interaction term of x-hour ambient air temperature and rainfall depth

* Actual terms used in the final model are centered.

**Table 3:** Initial predictive variables for input into the statistical models of near-surface air temperature.

Symbol	Description
u	Wind speed (m/s)
T	Ambient air temperature (°C)
$T_{surf}$	Surface temperature
SR	Solar radiation (in irradiance, kW/m^2^)
$T_{x-h}$	x-hour antecedent mean ambient air temperature (°C)
$R_{x-h}$	x-hour antecedent rainfall depth (mm)
$T_{x-h}\times R_{x-h}$	The interaction term of x-hour air temperature and rainfall depth

**Table 4:** Summary of data collected from the pervious site.

	Wind Speed (m/s)	Air Temp. (°C)	Relative Humidity (%)	Solar Radiation (kW/m^2^)	Surface Temp. (°C)	Air Temperature (°C)				
						0.05 m	0.5 m	1 m	2 m	3 m
Mean	**0.68**	**30.6**	**66**	**0.41**	39.7	33.4	32.6	32.6	32.5	32.5
95% CI	(0, 9.25)	(24.5, 37.0)	(47, 85)	(0, 0.95)	(26.0, 52.6)	(24.7, 42.4)	(24.4, 41.3)	(24.2, 40.2)	(24.1, 40.5)	(24.2, 40.4)

**Table 5:** Summary of data collected from the impervious site.

	Wind Speed (m/s)	Air Temp. (°C)	Relative Humidity (%)	Solar Radiation (kW/m^2^)	Surface Temp. (°C)	Air Temperature (°C)				
						0.05 m	0.5 m	1 m	2 m	3 m
Mean	**0.34**	**30.6**	**66**	**0.42**	42.0	33.8	32.8	32.8	32.6	32.7
95% CI	(0, 6.38)	(24.2, 36.9	(47, 84)	(0, 0.96)	(28.3, 56.4)	(24.8, 42.9)	(24.3, 40.6)	(24.1, 40.7)	(24.5, 40.3)	(24.9, 40.1)

**Table 6:** Statistical modeling results for standard least-squares models of near-surface air temperature ($T_{24h}$: 24-h antecedent mean ambient temperature, $R_{24h}$: 24-h antecedent rainfall depth).

Pervious Pavement Site							
Model Terms for 0.05 m Temp.	Coefficient	*p*-Value	95% CI	Model Terms for 0.5-m Temp.	Coefficient	*p*-Value	95% CI
$T_{24h}$	0.0044	0.95	(−0.15, 0.16)	$T_{24h}$	−0.00039	0.99	(−0.12, 0.12)
$R_{24h}$	−0.028	0.25	(−0.076, 0.020)	$R_{24h}$	−0.024	0.23	(−0.063, 0.015)
$T_{24h}\times R_{24h}$	−0.040	0.0044 *	(−0.067, −0.013)	$T_{24h}\times R_{24h}$	−0.031	0.0062 *	(−0.053, −0.0090)
Model Terms for 1 m Temp.	Coefficient	*p*-value	95% CI	Model Terms for 1.8 m Temp. ^+^	Coefficient	*p*-value	95% CI
$T_{24h}$	0.0031	0.96	(−0.12, 0.13)	$T_{24h}$	0.019	0.76	(−0.10, 0.14)
$R_{24h}$	−0.023	0.25	(−0.063, 0.017)	$R_{24h}$	−0.025	0.21	(−0.064, 0.014)
$T_{24h}\times R_{24h}$	−0.032	0.0063 *	(−0.054, −0.0092)	$T_{24h}\times R_{24h}$	−0.023	0.041 *	(−0.045, −0.00098)
Model Terms for 1.9 m Temp. ^+^	Coefficient	*p*−value	95% CI	Model Terms for 2 m Temp.	Coefficient	*p*−value	95% CI
$T_{24h}$	0.021	0.74	(−0.10, 0.15)	$T_{24h}$	0.023	0.71	(−0.10, 0.15)
$R_{24h}$	−0.025	0.21	(−0.064, 0.014)	$R_{24h}$	−0.025	0.20	(−0.064, 0.014)
$T_{24h}\times R_{24h}$	−0.022	0.052	(−0.044, 0.00016)	$T_{24h}\times R_{24h}$	−0.021	0.065	(−0.043, 0.0013)
Model Terms for 3 m Temp.	Coefficient	*p*-value	95% CI				
$T_{24h}$	0.031	0.63	(−0.094, 0.16)				
$R_{24h}$	−0.027	0.18	(−0.066, 0.013)				
$T_{24h}\times R_{24h}$	−0.018	0.12	(−0.040, 0.0044)				
Impervious Pavement Site							
Model Terms for 0.05 m Temp.	Coefficient	*p*-value	95% CI	Model Terms for 0.5 m Temp.	Coefficient	*p*-value	95% CI
$T_{24h}$	0.065	0.40	(−0.088, 0.22)	$T_{24h}$	0.043	0.47	(−0.076, 0.16)
$R_{24h}$	0.026	0.38	(−0.033, 0.086)	$R_{24h}$	0.023	0.32	(−0.023, 0.069)
$T_{24h}\times R_{24h}$	−0.033	0.028 *	(−0.063, 0.0037)	$T_{24h}\times R_{24h}$	−0.025	0.030 *	(−0.048, −0.0025)
Model Terms for 0.7 m Temp. ^+^	Coefficient	*p*-value	95% CI	Model Terms for 0.8 m Temp. ^+^	Coefficient	*p*-value	95% CI
$T_{24h}$	0.057	0.35	(−0.062, 0.18)	$T_{24h}$	0.063	0.29	(−0.056, 0.18)
$R_{24h}$	0.023	0.32	(−0.023, 0.069)	$R_{24h}$	0.023	0.33	(−0.023, 0.069)
$T_{24h}\times R_{24h}$	−0.023	0.047 *	(−0.046, 0.00030)	$T_{24h}\times R_{24h}$	−0.022	0.059	(−0.045, 0.00089)
Model Terms for 1 m Temp.	Coefficient	*p*-value	95% CI	Model Terms for 2 m Temp.	Coefficient	*p*-value	95% CI
$T_{24h}$	0.077	0.21	(−0.044, 0.20)	$T_{24h}$	0.071	0.25	(−0.049, 0.19)
$R_{24h}$	0.023	0.34	(−0.024, 0.070)	$R_{24h}$	0.021	0.38	(−0.026, 0.067)
$T_{24h}\times R_{24h}$	−0.020	0.093	(−0.043, 0.0034)	$T_{24h}\times R_{24h}$	−0.017	0.17	(−0.039, 0.0069)
Model Terms for 3 m Temp.	Coefficient	*p*-value	95% CI				
$T_{24h}$	0.064	0.29	(−0.055, 0.18)				
$R_{24h}$	0.022	0.34	−0.024, 0.068)				
$\;T_{24h}\times R_{24h}$	−0.017	0.14	(−0.040, 0.0058)				

* Statistically significant. ^+^ Temperature interpolated.

**Table 7:** Details of coefficients of statistical models for surface temperature.

	Pervious Surface (R^2^ = 0.81)				Impervious Surface (R^2^ = 0.84)			
Term	*p*-Value	Coefficient	95% CI	VIF	*p*-Value	Coefficient	95% CI	VIF
T	0.0005 *	1.22	(0.55, 1.88)	8.04	<0.0001 *	1.25	(0.67, 1.83)	8.42
SR	<0.0001 *	12.56	(8.27, 16.86)	2.59	<0.0001 *	11.37	(7.76, 14.98)	2.70
$T_{24h}$	0.36	−0.27	(-0.85, 0.31)	5.94	0.76	−0.080	(−0.61, 0.45)	6.75
$R_{24h}$	0.31	−0.043	(−0.13, 0.040)	1.06	0.74	0.015	(−0.072, 0.10)	1.22
$T_{24h}\times R_{24h}$ ^+^	0.0004 *	−0.086	(−0.13, −0.040)	1.19	0.0012 *	−0.071	(−0.11, −0.029)	1.30

* Statistically significant. ^+^ In centered form in the final model.

**Table 8:** Statistical models for the near-surface air temperature at the pervious site at different altitudes.

Altitude	Term	Intercept	u	T	$T_{surf}$	SR	$T_{24h}$
0.05 m (R^2^ = 0.95)	*p*-value	0.076	0.090	<0.0001 *	<0.0001 *	0.11	0.058
	Coeff.	−2.20	−0.095	1.00	0.23	1.26	−0.17
	95% CI	(−4.64, 0.24)	(−0.21, 0.015)	(0.79, 1.22)	(0.17, 0.30)	(−0.29, 2.81)	(−0.35, 0.0062)
	VIF	-	1.06	9.40	4.30	3.61	5.83
0.5 m (R^2^ = 0.95)	*p*-value	0.13	-	<0.0001 *	<0.0001 *	0.11	0.085
	Coeff.	−1.72	-	1.02	0.17	1.15	−0.14
	95% CI	(−3.94, 0.50)	-	(0.82, 1.22)	(0.11, 0.23)	(−0.27, 2.58)	(−0.30, 0.020)
	VIF	-	-	9.19	4.30	3.60	5.74
1 m (R^2^ = 0.94)	*p*-value	0.26	-	<0.0001 *	<0.0001 *	0.0018 *	-
	Coeff.	−1.42	-	0.89	0.15	2.20	-
	95% CI	(3.92, 1.07)	-	(0.78, 1.00)	(0.084, 0.21)	(0.85, 3.55)	-
	VIF	-	-	2.38	4.28	2.55	-
2 m (R^2^ = 0.95)	*p*-value	0.084	-	<0.0001 *	0.0004 *	0.018 *	0.065
	Coeff.	−2.03	-	1.10	0.11	1.81	−0.16
	95% CI	(−4.35, 0.28)	-	(0.90, 1.31)	(0.053, 0.17)	(0.33, 3.29)	(−0.33, 0.0098)
	VIF	-	-	9.19	4.30	3.60	5.74
3 m (R^2^ = 0.95)	*p*-value	0.086	-	<0.0001 *	0.0025 *	0.0052 *	0.064
	Coeff.	−2.02	-	1.13	0.094	2.14	−0.16
	95% CI	(−4.33, 0.29)	-	(0.92, 1.33)	(0.034, 0.15)	(0.66, 3.62)	(−0.33, 0.0093)
	VIF	-	-	9.19	4.30	3.60	5.74

* Statistically significant.

**Table 9:** Statistical models for the near-surface air temperature at the impervious site at different altitudes.

Altitude	Term	Intercept	T	$T_{surf}$	SR	$T_{24h}$	$R_{24h}$
0.05 m (R^2^ = 0.97)	*p*-value	<0.0001 *	<0.0001 *	<0.0001 *	0.071	0.0008 *	0.0045 *
	Coeff.	−4.16	1.16	0.23	1.22	−0.27	0.036
	95% CI	(−6.16, −2.16)	(0.97, 1.35)	(0.16, 0.29)	(−0.10, 2.54)	(−0.43, −0.12)	(0.011, 0.060)
	VIF	-	9.73	5.58	4.05	6.41	1.08
0.5 m (R^2^ = 0.97)	*p*-value	0.0007 *	<0.0001 *	<0.0001 *	0.0046 *	0.016 *	0.0066 *
	Coeff.	−2.85	1.06	0.16	1.57	−0.15	0.028
	95% CI	(−4.47, −1.23)	(0.91, 1.22)	(0.11, 0.21)	(0.50, 2.64)	(−0.28, −0.029)	(0.0080, 0.048)
	VIF	-	9.73	5.58	4.05	6.41	1.08
1 m (R^2^ = 0.97)	*p*-value	<0.0001 *	<0.0001 *	0.0001 *	0.0097 *	0.026 *	0.0089 *
	Coeff.	−3.92	1.16	0.12	1.67	−0.17	0.031
	95% CI	(−5.81, −2.02)	(0.98, 1.34)	(0.063, 0.18)	(0.42, 2.93)	(−0.31, −0.020)	(0.0081, 0.054)
	VIF	-	9.73	5.58	4.05	6.41	1.08
2 m (R^2^ = 0.96)	*p*-value	0.0004 *	<0.0001 *	0.0001 *	0.0306 *	0.020 *	0.011 *
	Coeff.	−3.65	1.16	0.13	1.44	−0.18	0.032
	95% CI	(−5.62, −1.68)	(0.97, 1.34)	(0.064, 0.19)	(0.14, 2.75)	(−0.33, −0.030)	(0.0077, 0.056)
	VIF	-	9.73	5.58	4.05	6.41	1.08
3 m (R^2^ = 0.96)	*p*-value	0.0019 *	<0.0001 *	<0.0001 *	0.0213 *	0.051	0.013 *
	Coeff.	−3.21	1.11	0.13	1.55	−0.15	0.031
	95% CI	(−5.20, −1.23)	(0.92, 1.30)	(0.068, 0.19)	(0.24, 2.87)	(−0.31, 0.00074)	(0.0066, 0.055)
	VIF	-	9.73	5.58	4.05	6.41	1.08

* Statistically significant.

## Data Availability

Data available on request from correspondence.
